# Oral health conditions in children with idiopathic nephrotic syndrome: a cross-sectional study

**DOI:** 10.1186/s12903-020-01197-1

**Published:** 2020-07-29

**Authors:** Urszula Kaczmarek, Alina Wrzyszcz-Kowalczyk, Katarzyna Jankowska, Katarzyna Prościak, Monika Mysiak-Dębska, Iwona Przywitowska, Irena Makulska

**Affiliations:** 1 Department of Conservative Dentistry and Pedodontics, Wroclaw Medical University, Krakowska 26, 50-425 Wroclaw, Poland; 2 Department and Clinic of Pediatric Nephrology, Wroclaw Medical University, Borowska 213, 50-556 Wroclaw, Poland

**Keywords:** Idiopathic nephrotic syndrome, Caries, Enamel hypoplasia, Oral hygiene, Gingival inflammation

## Abstract

**Background:**

Nephrotic syndrome is one of the chronic illnesses in the pediatric age group. The aim of this study was to assess the oral health of patients with steroid-sensitive idiopathic nephrotic syndrome (iNS).

**Methods:**

A case-control study was performed on iNS patients and healthy from May 2018 to April 2019. Dental caries was assessed by the World Health Organization criteria, developmental defects of enamel by the mDDE index, oral hygiene by the OHI-S and API, and gingival condition by the GI. Oral health behavior was recorded using a standardized questionnaire including tooth brushing, fluoride prevention, dietary habits and utilization of dental care. Additionally, Streptococcus mutans (SM) and Lactobacillus spp. (LB) bacteria in saliva were assessed using the CRT bacteria test. Statistical analysis comparing oral health parameters was carried by Pearson’s chi-squared, Fisher’s exact, Shapiro-Wilk verified by Student’s t or Mann-Whitney U tests.

**Results:**

The study included 94 participants of both sexes at the age of 4 to 17 years (47 cases and 47 controls) who were treated in Clinic of Pediatric Nephrology or outpatients’ dental clinic in Wroclaw, Poland. The iNS patients compared to the controls revealed some lower caries experience (83.0% vs 95.7%) and number of caries affected primary and/or permanent teeth (4.6 ± 3.5 vs 6.0 ± 4.1), a significantly lower number of filled primary and/or permanent teeth (1.1 ± 1.6 vs 3.5 ± 3.0, *P* < 0.001) and higher incidence of enamel hypoplasia (31.9% vs 4.3%, P < 0.001). The numbers of the iNS patients with high level of SM and LB were similar to the controls. The iNS patients had a higher OHI-S score (1.89 ± 1.59 vs 1.05 ± 1.02, *P* < 0.010) and a higher GI score (0.7 ± 1.0 vs 0.3 ± 0.6, *P* = 0.050). Moreover, they less frequently brushed their teeth twice a day (78.8% vs 93.6%, *P* = 0.026) and more frequently consumed three or more snacks daily (53.2% vs 23.4%, *P* = 0.002).

**Conclusions:**

The iNS patients despite the poor oral hygiene revealed lower caries experience but smaller number of restored caries-affected teeth, more severe gingivitis and more frequently teeth affected by enamel hypoplasia.

## Background

Nephrotic syndrome is a common chronic glomerular disease in childhood. Primary causes of the syndrome include minimal change disease, focal segmental glomerulosclerosis, membranous nephropathy, genetic disorders and secondary diseases linked to infections, drugs and neoplasia; however, it can also be idiopathic [1]. It presents itself as a combination of clinical and biochemical symptoms resulting from proteinuria, hypoproteinemia, hypoalbuminemia, hyperlipoproteinemia and an increase in cholesterol and triglycerides. Clinical symptoms include edema of the legs and hands or face, weight gain, feeling very tired, not feeling hungry and foamy or bubbly urine. The disease affects children of any age, from infancy to adolescence, however, predominantly between 1 and 6 years of age [2, 3]. The worldwide prevalence of idiopathic nephrotic syndrome is approximately 16 cases per 100,000 children with an incidence of 2 to 7 per 100,000 children [1–3].

Nephrotic syndrome is classified by response or lack of response to a standardized corticosteroid therapy into steroid-sensitive and steroid-resistant types. The majority of patients are treated by corticosteroids administration and 80% of children respond to corticosteroid therapy. However, even steroid-sensitive types of the disease can have a frequently relapsing course, requiring administration of alternative immunosuppressive agents. Long-term prognosis for steroid-sensitive diseases is excellent, but steroid-resistant ones constitute a future risk of chronic or end-stage renal disease [4, 5]. The frequent steroids administration may affect oral health adversely leading to candidiasis as well as an impairment of bone metabolism causing a considerable decrease in the mandibular bone mineral density [6–8].

The protocol of nephrotic syndrome therapy, except steroids, includes specific dietary regimes with a limitation of sodium, diuretics if edema and ascites, antibiotics in the case of infection. The prolonged administration of antibiotics (especially amoxicillin) can cause disturbances of hard dental tissues development, leading to defects of the enamel [9, 10]. In young children with chronic renal disease, which is frequently accompanied by altered mineral metabolism, developmental defects of enamel will occur in early postnatal life. Patients with nephrotic syndrome show disturbance of calcium homeostasis due to hypocalcemia, reduced vitamin D metabolites in serum, weakened intestinal absorption of calcium and raised level of parathyroid hormone, which lead to abnormal bone histology. The changes are assigned to the loss of various plasma proteins and minerals in urine, and steroid therapy [11, 12]. Disturbances in calcium, phosphorus and vitamin D metabolism occurring during the formation of enamel in developing teeth can result in developmental defects of enamel as demonstrated by other studies [13, 14].

The priority and permanent concern for the general health can prone to neglect of oral condition care; it can lead to irregular teeth cleaning and dental check-ups. An occasional removal of dental biofilm promotes to its accumulation and can predispose to gingival inflammation, drug-induced gingival hyperplasia and an increase of destructive periodontal diseases [6, 15]. However, the data on dental caries experience in young patients with nephrotic syndrome are scarce and inconsistent pointing on lower or greater caries occurrence [16, 17].

This study investigated selected oral health parameters in young participants suffering from idiopathic steroid-sensitive nephrotic syndrome (iNS) and compared to healthy ones. The null hypothesis was there was no difference in the studied parameters between two groups.

## Material and methods

### Study design

The study was an observational study comparing the oral condition in idiopathic steroid-sensitive nephrotic syndrome (iNS) patients treated in the Department and Clinic of Pediatric Nephrology with healthy outpatients attending to the dental clinic at the Department of Conservative Dentistry and Pedodontics of Wroclaw Medical University, Poland. The examinations were carried out from May 2018 to April 2019. The STROBE guidelines (Strengthening the Reporting of Observational Studies in Epidemiology) were followed [18].

### Participants

The enrolled participants (*n* = 110) both male and female, were at the age of 4 to 17 years. However, 9.1% (*n* = 10) of the parents did not express consent to the study and 5.4% (*n* = 6) of the sampled children refused to be examined. Therefore, 94 participants were finally included in the study. The patients with diagnosed idiopathic steroid-sensitive nephrotic syndrome (*n* = 47) in remission (*n* = 26) or relapse (*n* = 21) phases were selected from the pool of patients hospitalized in the Clinic of Pediatric Nephrology. The inclusive criteria were the disease lasting at least 2 years at the time of oral examination and no other acute systemic diseases currently present. The control group comprised clinically healthy participants (n = 47) with a negative history of renal disease and acute or chronic systemic diseases at the age range and sex corresponding to the iNS patients, who visited the outpatient dental clinic due to routine dental treatment or checkups. All participants involved in the study had to provide written informed consent of a parent (and consent of a patient at the age of 16 and over), a completed questionnaire, and cooperation during oral clinical examinations and the collection of saliva. The participants who did not fulfil the inclusion criteria were excluded from the study.

### Ethical permission

The study protocol was approved by the Bioethics Committee of Wroclaw Medical University (permission no. KB–343/2016) in accordance with the Declaration of Helsinki. Participation in the study was voluntary and anonymous, and the collected data was treated confidentially.

### Sample size estimation

Sample size determination was based on t-test for independent groups using a special computer program [19]. An expected difference between means for two groups for primary and/or permanent caries-affected teeth was set at 1.3 (variance equal to 5.0) because the previous study revealed such a difference [16]. Power of the test was set at 80% and confidence level at 95%. With such assumptions required sample size for each group was equal to *n* = 47.

### Oral examination

The dental examination was performed with the use of artificial light, a plane mirror and a ball-ended dental probe (WHO CPI probe). Decayed, missing and filled primary and permanent teeth (dmft, DMFT) were assessed according to the recommended criteria of the World Health Organization (WHO) [20]. Caries experience was expressed as the sum of caries-affected primary and permanent teeth because different number of participants in both iNS and control groups had exclusively primary (11 vs 10 participants), mixed (28 vs 22 participants) or permanent dentition (8 vs 15 participants, respectively). In order to evaluate oral hygiene, two indices were used - the Simplified Oral Hygiene Index (OHI-S) obtained by summation of Debri Index (DI) and Calculus Index (CI), by Green and Vermillion, 1964 [21] and the Approximal Plaque Index – API, by Lange et al., 1974 [22]. The used categorization of OHI-S scores was as follows: 0–1.2 good, 1.3–3.0 fair, 3.1–6.0 poor oral hygiene, and for DI-S and CI-S 0–0.6 good, 0.7–1.8 fair and 1.9–3.0 poor oral hygiene. The criteria of API value were: < 25% optimal, 25–39% quite good, 40–69% moderate and 70–100% poor oral hygiene. The periodontal condition was assessed according to the Gingival Index – GI by Löe and Silness, 1963 [22] which evaluates the severity of gingivitis, where the values in the range of 0.1–1.0 point on mild, 1.1–2.0 moderate and 2.1–3.0 severe gingivitis. Presence of enamel hypoplasia was scored according to the modified Developmental Defects of Enamel (mDDE) index [23]. No oral mucosal lesions were found in all participants.

The assessment of oral condition was carried out by examiners, being pediatric dentists (AW-K, KJ, MM-D, IP), that were calibrated by an experienced supervisor (UK) before the study (all of them have been working in the Department of Conservative Dentistry and Pedodontics). Cohen’s kappa scores for intra- and inter-examiner reliability were > 0.80 [24].

### Cariogenic bacteria assessment in saliva

In stimulated mixed saliva, counts of Streptococcus mutans (SM) and Lactobacillus spp. (LB) were assessed using a CRT bacteria test (Vivadent Ivoclar). Saliva stimulated by chewing paraffin pellet was collected in a plastic container followed by mouth rinsing with used of distilled water. The both agar carrier surfaces of the test were wet with saliva. The test vial was closed and placed in the incubator at 37 °C for 48 h. The density of SM and LB colonies were compared with model chart. Results of 10^5^ CFU (colony forming unit) or more of SM and LB indicate a high caries risk [25].

### Questionnaire

The designed questionnaire for this study contained 12 standard items on demographic and social background (gender, parent’s education, economic status), utilization of dental care, oral health related behaviors (frequency of tooth brushing, usage of fluoridated toothpaste and other fluoride products and consumption of sugary food and beverages (Supplementary file 1). Economic status was categorized in view of the total family income, the number of family members and the national average salary (Supplementary file 2).

### Statistical analysis

The obtained data were analyzed using Pearson’s chi-squared test or Fisher’s exact test for sociodemographic and practices variables. Normality was analyzed using the Shapiro-Wilk test and was verified using the Student’s t test (for independent variables) or the Mann-Whitney U test (for data with a non-normal distribution) along with the Spearman’s rank correlation coefficient with the help of Statistica v. 13.0 PL StatSoft software (StatSoft Poland). For all statistical tests, the significance level was set at *P* < 0.05. Cohen’s kappa statistics was used to determine the inter- and intra-examiner reliability in caries and enamel hypoplasia diagnosis, oral hygiene and gingival status assessment [24].

## Results

### Distribution of the participants

Ninety-four participants were enrolled to the study, half of them were with idiopathic steroid-sensitive nephrotic syndrome and the remainders were healthy. The disease was diagnosed between the ages of 4 and 15 years (mean 3.8 ± 3.0 years old), duration of the disease at the time of the study ranged from 2 to 15 years (mean 6.4 ± 4.0 years), and number of relapses from 1 to 15 (mean 3.8 ± 2.9).

The mean age in the iNS group was 9.6 ± 3.9 years and in the controls 10.8 ± 3.7 years, and did not differ significantly. Economic status of the family and number of children were similar in both groups. More mothers and fewer fathers of the iNS participants had secondary or higher level of education (Table 1).

**Table 1:** Characteristics of the studied participants

		iNS		Control		*P* value
		n	%	n	%	
Gender	male	25	53.2	18	38.3	0.147
	female	22	46.8	29	61.7	
Age	mean SD	9.6 ± 3.9		10.8 ± 3.7		0.142
Family economic status	high	4	8.5	10	21.3	0.164
	moderate	34	72.4	24	51.1	
	satisfactory	7	14.9	11	23.4	
	unsatisfactory	2	4.2	2	4.2	
Children’s number in the family	1	11	23.4	13	27.7	0.250
	2	21	44.7	26	55.3	
	3	11	23.4	4	8.5	
	4+	4	8.5	4	8.5	
Father’s education	higher	8	17.0	12	25.5	0.030*
	secondary	15	31.9	20	42.6	
	primary	24	51.1	15	31.9	
Mother’s education	high	11	23.4	19	40.4	0.029*
	secondary	25	53.2	18	38.3	
	primary	11	23.4	10	21.3	

*iNS* Idiopathic nephrotic syndrome

* - significant difference at *P* < 0.05

### Oral health parameters

Inter- and intra-examiner reproducibility of the oral clinical examination parameters, on the average, was 0.9 (Cohen’s kappa coefficient). The participants suffering from iNS compared to the controls revealed some lower caries experience (83.0% vs 95.7%) and a slightly smaller number of caries affected primary and/or permanent teeth (4.6 ± 3.5 vs 6.0 ± 4.1). The iNS patients had over a 3-fold lower number of filled teeth than healthy (*P* < 0.001) and similar numbers of decayed and missing primary and/or permanent teeth (Table 2).

**Table 2:** Oral health parameters

	iNS		Control		*P* value
Caries experience	n/N	%	n/N	%	0.070
	39/47	83.0	45/47	95.7	
	mean SD		mean SD		
Number of caries-affected primary and/or permanent teeth	4.6 ± 3.5		6.0 ± 4.1		0.126
primary and/or permanent decayed teeth	3.5 ± 3.2		2.4 ± 2.4		0.095
primary and/or permanent missing teeth	0.02 ± 0.1		0.2 ± 0.8		0.543
primary and/or permanent filled teeth	1.1 ± 1.6		3.5 ± 3.0		< 0.001***
DMFT	2.40 ± 3.04		4.21 ± 4.77		0.047*
DT	1.72 ± 2.71		1.47 ± 2.37		0.865
MT	0.02 ± 0.14		0.17 ± 0.81		0.851
FT	0.66 ± 0.65		2.57 ± 3.35		0.018*
dmft	2.19 ± 2.93		1.79 ± 2.35		0.561
dt	1.74 ± 2.25		0.89 ± 1.33		0.152
mt	0		0.02 ± 0.14		0.865
ft	0.45 ± 1.33		0.89 ± 1.43		0.136
	n	%	n	%	
Enamel hypoplasia frequency	15	31.9	2	4.3	< 0.001***
	mean SD		mean SD		
Number of teeth with enamel hypoplasia	0.80 ± 1.5		0.10 ± 0.3		< 0.001***
OHI-S	1.89 ± 1.59		1.05 ± 1.02		0.010**
DI-S	1.80 ± 1.55		0.99 ± 0.92		0.011*
CI-S	0.09 ± 0.31		0.06 ± 0.26		0.463
API (%)	54.0 ± 35.7		43.4 ± 27.6		0.108
GI	0.7 ± 1.0		0.3 ± 0.6		0.050
CFU Streptococcus mutans (SM)					
< 10^5^	36	76.6	39	83.0	0.441
≥ 10^5^	11	23.4	8	17.0	
CFU Lactobacilli spp. (LB)					
< 10^5^	28	59.6	27	57.4	0.834
≥ 10^5^	19	40.4	20	42.6	

*Abbreviations: dmft* Number of decayed, missing due to caries and filled primary teeth; *DMFT* Number of decayed, missing due caries and filled permanent teeth, *OHI-S* Simplified Oral Hygiene Index, *DI* Debris index, *CI* Calculus index, *API* Approximal plaque index, *GI* Gingival index, *CFU* Colony forming unit, *iNS* Idiopathic nephrotic syndrome

*- significant difference at *P* < 0.05

**- significant difference at *P* < 0.01

*** - significant difference at *P* < 0.001

Regarding caries-affected permanent teeth, significantly lower DMFT and FT values were found in iNS patients than the controls (*P* = 0.047 and *P* = 0.018, respectively). However, there was no significant difference in caries experience of primary teeth between the two groups (Table 2). In the iNS group, the number of caries-affected teeth (dmft/DMFT) was positively moderately correlated with a DI-OHI-S value (r = 0.337, *P* = 0.021). While in healthy participants, a significant moderate correlation between DI-OHI-S and dt/DT values (r = 0.425, *P* = 0.003) was noticed [26].

Among the various types of developmental defects of enamel, only hypoplastic lesions in the examined participants were noticed. The prevalence of enamel hypoplasia was over 7 times higher in the iNS compared to the control group (P < 0.001) and, on average, 8-fold more teeth were affected (*p* < 0.001) (Table 2).

**Fig. 1:**
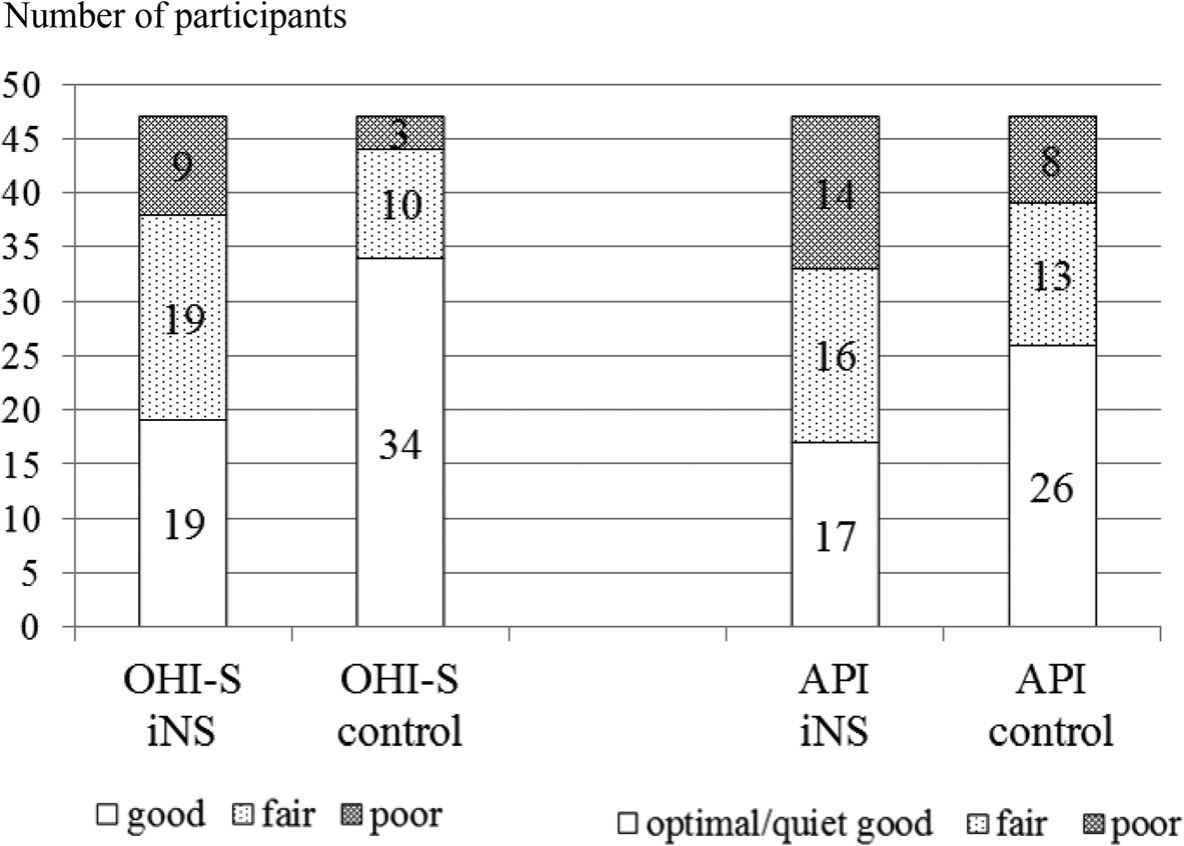
Distribution of participants with oral hygiene levels according to OHI-S and API indices

As for oral hygiene, a higher mean of OHI-S and DI-S scores was found in the iNS group compared to the control group; however, API values were only slightly higher (Table 2). Analysis of distribution of oral hygiene levels showed that 19 iNS participants (40.4%) and 34 controls (72.3%) had good oral hygiene according to OHI-S criteria, 17 (36.2%) and 26 (55.3%) optimal or quite good by API scores (Fig. 1). In both groups API values were positively moderately correlated with the number of caries-affected teeth (r = 0.371, *P* = 0.010 and r = 0.398, *P* = 0.005).

**Fig. 2:**
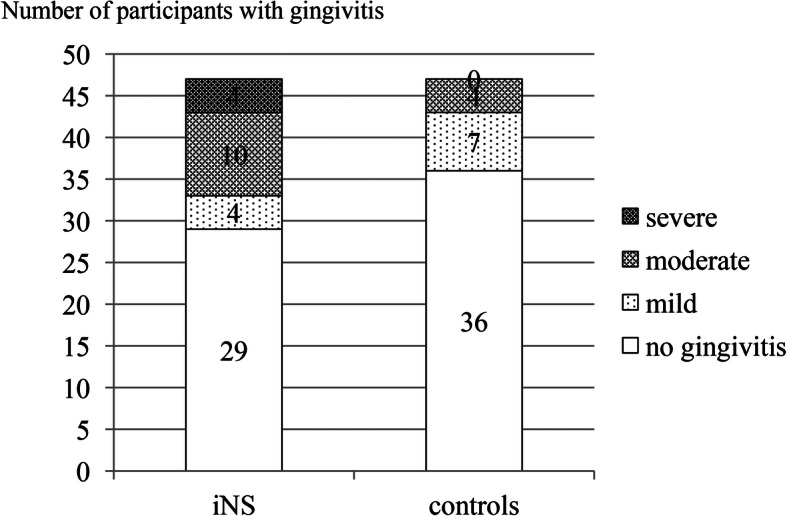

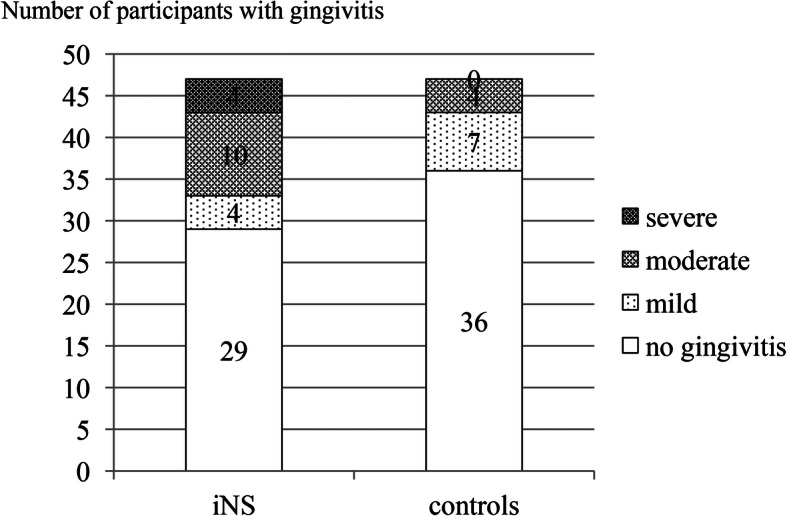
Distribution of participants with gingivitis

Regarding gingival condition a 2-fold higher mean GI value was found in the iNS patients compared to the controls (*P* = 0.050). However, in both groups the mean GI scores were within the limit of mild gingivitis (Table 2). Analysis of GI values among the studied participants showed that 29 iNS patients (61.7%) had clinically sound gingival tissues, 4 (8.5%) mild, 10 (21.3%), moderate and 4 (8.5%) severe gingivitis. In the controls the figures were 36 (76.6%), 7 (14.9%), 4 (8.5%) and zero, respectively (Fig. 2). In the iNS group, GI values were positively moderately correlated with DI-S and API values (r = 0.456, *P* = 0.001, r = 0.341, *P* = 0.018, respectively), however, in the control group only with DI-S (r = 0.393, *P* = 0.006; positive moderate correlation).

The percentage of participants with low and high count levels of Streptococcus mutans and Lactobacillus spp. in saliva did not differ significantly between the iNS and control groups (Table 2).

Regarding oral hygiene practice, significantly fewer iNS participants brushed their teeth twice a day compared to the controls; however, both groups used fluoridated toothpastes with similar frequency. There was no difference between the study groups in terms of the last dental visit and its cause. The iNS participants more frequently consumed three or more snacks per day than the controls (56.3% vs 23.4%, *P* = 0.002) but both ingested sweets and sweetened beverages with similar frequency (Table 3).

**Table 3:** Oral hygiene, dietary habits and utilization of dental care

Groups	iNS		Control		*P* value
	n	%	n	%	
Tooth brushing					
Twice a day	37	78.8	44	93.6	0.026*
Once a day or every few days	10	21.2	3	6.4	
Dentifrice					
Fluoridated	43	91.5	42	89.4	0.603
No fluoride	4	8.5	5	10.6	
Frequency of sweets consumption					
Several times a day	11	23.4	13	27.7	0.401
Once a day	20	42.6	21	44.7	
Once a week	10	21.3	12	25.5	
Once a month	6	12.7	1	2.1	
Frequency of drinking sweetened beverages					
Several times a day	13	27.6	11	23.4	0.312
Once a day	11	23.4	14	29.8	
Once a week	4	8.5	7	14.9	
Once a month	19	40.5	15	31.9	
Number of snacks a day					
One	4	8.5	2	4.3	0.002*
Two	18	38.3	34	72.3	
Three and more	25	53.2	11	23.4	
Last dental visit					
Within 6 months	36	76.6	42	89.4	0.204
12 months	9	19.1	5	10.6	
Does not remember	2	4.3	0	0	
Cause of last dental visit					
Pain	6	12.8	0	0	0.901
Decay	17	36.2	23	48.9	
Control	19	40.4	19	40.5	
Other cause	5	10.6	5	10.6	
Topical professional application of fluoride specimens					
Yes	16	34.0	17	36.2	0.541
No or does know	31	66.0	30	63.8	

*Abbreviation*: *iNS* Idiopathic nephrotic syndrome

*- significant difference at *P* < 0.05

**- significant difference at *P* < 0.001

## Discussion

The null hypothesis was rejected, as there were differences in the studied oral parameters between two groups. This study showed some lower caries experience in the iNS participants compared to healthy ones (83.0% vs 95.7%). Like Güzel et al. [17] in pediatric patients with nephrotic syndrome in remission phase, we found some lower dmft and DMFT values in iNS patients compared to the controls. On the contrary, another study presented significantly higher dmft and slightly lower DMFT values compared to the controls [16]. The higher number of unfilled tooth decays in the iNS patients compared to the controls could be the result of the neglect of dental condition due to continuous care of the general health of children as significantly fewer of them brushed their teeth twice a day.

The number of iNS and healthy participants with high levels of Streptococcus mutans and Lactobacillus spp. did not differ in our study, which could point to a similar caries risk. Other study presented significantly less frequent the isolation of Streptococcus mutans from children with chronic renal failure compared with the controls [14]. Contrary, Takeuchi et al. [27] assessing cariogenic bacteria with use of Dentocult test in patients with renal disease found a significantly higher bacterial counts compared to healthy controls and suggested a higher caries risk development in the patients.

The significantly worse oral hygiene in the iNS group compared to the controls based on the mean score of OHI-S in our study was found. Another study assessing oral hygiene with use of Plaque Index - PLI (by Silness-Lȍe) could confirm our finding as significantly higher score value in children suffering from nephrotic syndrome compared to the healthy ones was noticed [6].

Deterioration of defense mechanisms in nephrotic syndrome results from hyperlipidemia and immunosuppressive impact of taken medications, including steroids. All children from India suffering from nephrotic syndrome presented inflammation of gingival tissues, assessed by the Modified Gingival Index by Lobene. Contrarily, our data based on the Gingival Index (by Lőe and Silness) showed clinically healthy gingiva in 29 (61.7%) of iNS participants. Our iNS patients revealed some higher mean GI scores compared to the control group, unlike other results, where the difference was significant [6]. Angelova [28] found over a 2-fold higher GI value compared to our data (1.59 vs 0.7).

Gingival inflammation is caused by dental plaque accumulation due to poor oral hygiene, which confirmed our data. Attention directed to medical care, frequent hospitalization resulting from relapses of the disease and hypoplastic teeth leaning towards dental biofilm accumulation could be the causes of higher dental plaque scores in the iNS patients. However, immunosuppression associated with treatment of nephrotic syndrome may alter the inflammatory response of gingival tissue to bacterial plaque. In addition, gingivitis may mask the pallor caused by anemia being a systemic manifestation of altered renal function [29].

The age at which the first disease episode occurs, correlates with the abnormality development of emerging dental enamel at the time. We found that the iNS onset in 43 patients occurred between 2 and 8 years of age, i.e. within the period of enamel formation in permanent teeth, which lasts approximately from 3 (first molars) to 7–8 years (second molars) when dental crowns (enamel) are completed [30]. Therefore, enamel disturbances could develop in these patients; however, we noticed enamel hypoplasia in 15 participants. Nevertheless, enamel hypoplasia occurred approximately seven times more often in the patients with nephrotic syndrome than in the controls, and the defects involved eight times more teeth. Bublitz et al. [31] observed enamel hypoplasia in 20% of patients with steroid-sensitive nephrotic syndrome, which was lower compared to our result (31.9%). However, another study showed that only 1 out of 38 patients (i.e. 2.6%) was affected by enamel hypoplasia [17].

Long-term effective measures of dental plaque control should be implemented to avoid the risk of caries development and gingival inflammation. Early diagnosis and prompt management of carious lesions are compulsory to avoid extensive dental treatment and potential infection foci from the oral cavity. Additionally, treatment of enamel hypoplasia should be performed when severe defects are present due to dental plaque retention and esthetics.

Collaboration between dentists and pediatric nephrologists, and better understanding of the interrelationship between systemic and oral abnormalities are required in dental care of patients with nephrotic syndrome. The dentist should also consider the adverse side effects of drug therapy and appropriate prescribing medications, in view of compromised renal function. Considering the chronic course of the disease, maintenance of oral health is important. In the majority of the iNS patients, the disease does not complicate dental care. The dental treatment of patients in remission period of the disease can be carried out regularly at that time. However, surgical procedures should be performed with suitable antibiotic cover and consultation with the nephrologist [32, 33].

The present study has some limitations and the results should be interpreted in the context of its design. It included a small number of participants who represented only a fraction of the total number of pediatric patients with idiopathic nephrotic syndrome and self-reported by parents’ questionnaires concerning factors influencing oral health. The strength was that calibrated dentists examined the participants and a control group of healthy participants was included.

The future prospective study seems to be needed to elucidate an impact of the disease and its treatment on oral health condition of the patients with respect to modifiable risk factors for oral diseases that act locally.

## Conclusions

The iNS patients despite the poor oral hygiene revealed lower caries experience but smaller number of restored caries-affected teeth, more severe gingivitis and more frequently teeth affected by enamel hypoplasia compared to the controls.

## Supplementary information

**Additional file 1: Supplementary file 1.** Table 3 Oral hygiene, dietary habits and utilization of dental care. Description of data: How often your child brushes his/her teeth: twice a day; once a day; every few days. Does your child use fluoridated toothpaste: yes, not. How often your child eats sweets (e.g. candies, chocolate, chocolate bar, cakes, sweet bun, donut, chips): several times a day, once a day, once a week, once a month. How often your child drinks sweetened beverages (e.g. coca-cola, pepsi cola, fanta, lemonade, tea beverages with added sugar). How many snacks your child eats a day: one, two three and more. When was last dental visit of your child: within last six months; 12 months ago, I do not remember. What was a cause of the last dental visit of your child: a tooth pain, tooth decay, control, others (e.g. continuation of dental treatment, unaesthetic appearance of teeth). Did your child had application of fluoride varnish, gel or foam in a dental office; yes, no, I do not know.

**Additional file 2: Supplementary file 2.** Table 1 Characteristics of the studied participants. Description of data: Children’ number of in the family: 1, 2, 3, 4, 5 or more, specify and mark. Family economic status*: ≥ 2001 zloty (high), 1693–2000 zloty (moderate), 1000–1692 zloty (satisfactory), ≤ 999 zloty (unsatisfactory). Father education (years of schooling): 10 (primary), 14–15 (secondary), 16–20 (high). Mother education (years of schooling): 10 (primary), 14–15 (secondary), 16–20 (high). *Family economic status was calculated based on the division of total family monthly income in zloty (father and mother) by number of the family members in respect to a national average income per family member, which was 1693 PLN in 2018.

## Data Availability

The datasets used and analyzed during the current study are available from the corresponding author on request.

## Abbreviations

DMFT: Decay, missing and filled permanent teeth
dmft: Decay, missing and filled primary teeth
mDDE: Modified Developmental Defects of Enamel
OHI-S: Simplified Oral Hygiene Index
DI: Debri Index
CI: Calculus Index
API: Approximal Plaque Index
PLI: Plaque Index
GI: Gingival Index
SM: Streptococcus mutans
LB: Lactobacillus spp
CFU: Colony forming unit
eGRF: Estimated glomerular filtration rate
WHO: World Health Organization
iNS: Idiopathic nephrotic syndrome
