# Supplementary material for: Oral health conditions in children with idiopathic nephrotic syndrome: a cross-sectional study
Source: BMC Oral Health. 2020 Jul 29;20:213. doi: 10.1186/s12903-020-01197-1 (PMC7391815; doi:10.1186/s12903-020-01197-1)
Supplement: Supplementary file 1 — Additional file 1: Supplementary file 1. Table 3 Oral hygiene, dietary habits and utilization of dental care. Description of data: How often your child brushes his/her teeth: twice a day; once a day; every few days. Does your child use fluoridated toothpaste: yes, not. How often your child eats sweets (e.g. candies, chocolate, chocolate bar, cakes, sweet bun, donut, chips): several times a day, once a day, once a week, once a month. How often your child drinks sweetened beverages (e.g. coca-cola, pepsi cola, fanta, lemonade, tea beverages with added sugar). How many snacks your child eats a day: one, two three and more. When was last dental visit of your child: within last six months; 12 months ago, I do not remember. What was a cause of the last dental visit of your child: a tooth pain, tooth decay, control, others (e.g. continuation of dental treatment, unaesthetic appearance of teeth). Did your child had application of fluoride varnish, gel or foam in a dental office; yes, no, I do not know. [file 12903_2020_1197_MOESM1_ESM.docx]

**Supplementary file 1**

Description of data (according to data from Table 3):

- **How often your child brushes his/her teeth?**

***answers:* twice a day; once a day; every few days**

- **Does your child use fluoridated toothpaste?**

***answers:* yes, no**

- **How often your child eats sweets (e.g. candies, chocolate, chocolate bar, cakes, sweet bun, donut, chips)?**

***answers:* several times a day, once a day, once a week, once a month**

- **How often your child drinks sweetened beverages (e.g. coca-cola, pepsi cola, fanta, lemonade, tea beverages with added sugar)?**

***answers:* several times a day, once a day, once a week, once a month**

- **How many snacks your child eats a day?**

***answers:* one, two three and more**

- **When was last dental visit of your child?**

***answers:* within last six months; 12 months ago, I do not remember**

- **What was a cause of the last dental visit of your child?**

***answers:* a tooth pain, tooth decay, control, others (e.g. continuation of dental treatment, unaesthetic appearance of teeth)**

- **Did your child had application of fluoride varnish, gel or foam in a dental office?**

***answers:* yes, no, I do not know**
