# Supplementary material for: Oral health conditions in children with idiopathic nephrotic syndrome: a cross-sectional study
Source: BMC Oral Health. 2020 Jul 29;20:213. doi: 10.1186/s12903-020-01197-1 (PMC7391815; doi:10.1186/s12903-020-01197-1)
Supplement: Supplementary file 2 — Additional file 2: Supplementary file 2. Table 1 Characteristics of the studied participants. Description of data: Children’ number of in the family: 1, 2, 3, 4, 5 or more, specify and mark. Family economic status*: ≥ 2001 zloty (high), 1693–2000 zloty (moderate), 1000–1692 zloty (satisfactory), ≤ 999 zloty (unsatisfactory). Father education (years of schooling): 10 (primary), 14–15 (secondary), 16–20 (high). Mother education (years of schooling): 10 (primary), 14–15 (secondary), 16–20 (high). *Family economic status was calculated based on the division of total family monthly income in zloty (father and mother) by number of the family members in respect to a national average income per family member, which was 1693 PLN in 2018. [file 12903_2020_1197_MOESM2_ESM.docx]

**Supplementary file 2**

Description of data (according to data from Table 1):

- Children’ number of in the family: 1, 2, 3, 4, 5 or more, specify and mark
- Family economic status*: ≥ 2001 zloty (high), 1693-2000 zloty (moderate), 1000-1692 zloty (satisfactory), ≤ 999 zloty (unsatisfactory)
- **Father education (years of schooling): 10 (primary), 14-15 (secondary), 16-20 (high)**
- **Mother education (years of schooling): 10 (primary), 14-15 (secondary), 16-20 (high)**

*Family economic status was calculated based on the division of total family monthly income in zloty (father and mother) by number of the family members in respect to a national average income per family member, which was 1693 PLN in 2018
